# Burden of polycystic ovary syndrome in the Middle East and North Africa region, 1990–2019

**DOI:** 10.1038/s41598-022-11006-0

**Published:** 2022-04-29

**Authors:** Kimia Motlagh Asghari, Seyed Aria Nejadghaderi, Mahasti Alizadeh, Sarvin Sanaie, Mark J. M. Sullman, Ali-Asghar Kolahi, Jodie Avery, Saeid Safiri

**Affiliations:** 1 Social Determinants of Health Research Center, Department of Community Medicine, Faculty of Medicine, Tabriz University of Medical Sciences, Tabriz, Iran; 2 School of Medicine, Shahid Beheshti University of Medical Sciences, Tehran, Iran; 3 Systematic Review and Meta-Analysis Expert Group (SRMEG), Universal Scientific Education and Research Network (USERN), Tehran, Iran; 4 Aging Research Institute, Tabriz University of Medical Sciences, Tabriz, Iran; 5 Department of Life and Health Sciences, University of Nicosia, Nicosia, Cyprus; 6 Department of Social Sciences, University of Nicosia, Nicosia, Cyprus; 7 Social Determinants of Health Research Center, Shahid Beheshti University of Medical Sciences, Tehran, Iran; 8 Adelaide Medical School, The University of Adelaide, Adelaide, South Australia Australia; 9 Robinson Research Institute, The University of Adelaide, Adelaide, South Australia Australia; 10 Women’s Reproductive Health Research Center, Tabriz University of Medical Sciences, Tabriz, Iran

**Keywords:** Endocrine reproductive disorders, Epidemiology

## Abstract

Polycystic ovary syndrome (PCOS) is one of the most important contributing factors to infertility. In this study, we report the burden of PCOS by age and sociodemographic index (SDI) for the 21 countries in the Middle East and North Africa (MENA) region. Publicly available data on the point prevalence, incidence and years lived with disability (YLDs), from 1990 to 2019, were retrieved from the Global Burden of Disease (GBD) 2019 study for the 21 countries in MENA. The results are presented with age-standardised numbers and rates per 100,000 population, along with their corresponding 95% uncertainty intervals (UIs). In 2019, the age-standardised point prevalence and incidence rate for PCOS in the MENA region were 2079.7 (95% UI: 1392.0 to 2812.3) and 77.2 (95% UI: 51.6 to 105.4) per 100,000, respectively, which represents a 37.9% (95% UI: 31.7 to 45.0) and a 33.7% (95% UI: 27.7 to 40.3) increase since 1990, respectively. Also in 2019, the age-standardised YLD rate of PCOS in this region was 18.7 (95% UI: 7.8 to 37.9) per 100,000 women, which has increased by 36.1% (95% UI: 29.4 to 43.4) since 1990. Kuwait [25.4 (10.7, 51.2)] had the highest age-standardised YLD rate, while Afghanistan [10.8 (10.1, 49.2)] had the lowest. Moreover, the largest increase in the YLD rate, from 1990 to 2019, was seen in Sudan [90.3% (64.1, 120.9)], whereas no country decreased during the measurement period. The total prevalent number and point prevalence of PCOS (per 100,000) were both highest in the 20–24 age group. The prevalence of PCOS was highest among women of reproductive age, but decreased rapidly after 45 years of age. Moreover, at the country level there was a positive association between SDI and the age-standardised YLD rates of PCOS. The growing prevalence and burden of PCOS in the MENA region highlights the need to implement cost-effective preventive programs, especially for women in their third decade of life, and in MENA countries with higher SDI levels.

## Introduction

Polycystic ovary syndrome (PCOS) is a chronic and heterogeneous disorder of the endocrine system, which manifests itself as menstrual dysfunction, infertility, hirsutism, acne and obesity^1^. Previous research has shown that both genetic and environmental factors are associated with the negative sequelae of PCOS, including obesity and infertilty^2,3^. PCOS is usually only diagnosed when complications develop that significantly reduce the patient’s quality of life (e.g., hair loss, acne, alopecia and infertility-related problems)^4^.

The global prevalence of PCOS is estimated to be 8–21% of women of reproductive age^5^. This high prevalence, and its association with ovulation and menstrual disorders, infertility, hair loss, and metabolic complications, highlights the large financial burden caused by PCOS^6,7^. Although PCOS can occur at any age, from menarche onwards, most cases are diagnosed between the ages of 20 and 30 years old^8^. Globally, 1.55 million women of reproductive age experience PCOS, resulting in 0.43 million disability-adjusted life-years (DALYs). In 2017, the age-standardised incidence rate of PCOS, among women of reproductive age, was 82.44 per 100,000 population, which was 1.45% higher than in 2007^9^.

Given the substantial number of women who suffer from PCOS, and its significant impact on the patient, a better understanding of the current burden in the Middle East and North Africa (MENA) region is important. However, other than the Global Burden of Disease (GBD) project, no studies have reported the burden of PCOS at the global, regional and national levels, or its relationship with socioeconomic status^9^. Furthermore, most epidemiological studies on PCOS have been conducted in developed countries, with only limited information available on the burden in developing countries^10,11^. To our best of knowledge, no study specifically conducted on the burden of PCOS in the MENA region using data of GBD studies. It is also important to note that the number of countries with available data increased from 23 in GBD 2017 to 30 in GBD 2019, which helped stimulate the present study.

The findings of this study could be used to inform policy decisions regarding the allocation of public health resources or the development and implementation of interventions. Any interventions must also taking into consideration the specific socio-cultural context and the economic situation in the MENA countries, where the prevalence of PCOS is high^12^. In this study, we report the prevalence, incidence and YLDs due to PCOS, by age and sociodemographic index (SDI), for the 21 countries and territories in MENA.

## Methods

### Overview

The Institute of Health Metrics and Evaluation (IHME) conducts the GBD studies, which measure the epidemiological levels and trends associated with communicable diseases, non-communicable diseases and injuries across the world. The last GBD iteration (i.e. GBD 2019) collected data on the burden of 369 diseases and injuries and 87 risk factors, from 1990 to 2019, in 204 countries and territories, seven super-regions and 21 regions^13,14^. PCOS is one of the most frequently studied diseases and its burden has been estimated for all regions across the world^9,13^. In the present study, we report the burden of PCOS for the countries that comprise the MENA region, from 1990 to 2019. The countries that comprise the MENA region include: Afghanistan, Algeria, Bahrain, Egypt, Iran (Islamic Republic of), Iraq, Jordan, Kuwait, Lebanon, Libya, Morocco, Oman, Palestine, Qatar, Saudi Arabia, Sudan, the Syrian Arab Republic, Tunisia, Turkey, the United Arab Emirates and Yemen. The general methodology for estimating the burden of diseases, injuries and risk factors for GBD 2019 has been reported in previous publications^13–15^ and the data is available at https://vizhub.healthdata.org/gbd-compare/ and http://ghdx.healthdata.org/gbd-results-tool.

The present study was approved by the ethics committee of the Tabriz University of Medical Sciences (IR.TBZMED.REC.1400.596). All methods were performed in accordance with the national guidelines and regulations.

### Case definition and data sources

PCOS is the most common endocrinopathy affecting women of reproductive age and is characterized by hyperandrogenism, ovulatory dysfunction, and polycystic ovaries. Some women with PCOS have enlarged ovaries that contain underdeveloped follicles, which appear as pockets of fluid or “follicular cysts”. In a review of PCOS symptoms from 12 trials, they reported that only 28% of women with PCOS exhibited polycystic ovaries^5^. PCOS symptoms include infrequent menstruation, excess hair growth, acne and obesity^16^, but there is no generally accepted definition of PCOS^17^. However, there are diagnostic criteria which have been developed by experts, including the National Institutes of Health (NIH) criteria^18^, the Rotterdam criteria^18^, and the criteria provided by the Androgen Excess and PCOS Society (AE-PCOS)^19^. All three diagnostic criteria require that all potential secondary causes are firstly ruled out, such as congenital adrenal hyperplasia, hyperprolactinemia and androgen-secreting neoplasms. Furthermore, all three diagnostic criteria require the presence of two or more signs or symptom of PCOS.

Although a diagnosis of PCOS can be made using any of the three diagnostic approaches (NIH, Rotterdam or AE-PCOS), the GBD 2019 used the NIH criteria developed by the American College of Obstetricians and Gynecologists (ACOG)^16^. The other two definitions (i.e., Rotterdam and AES) include more mild phenotypes, which can result in substantially higher prevalence estimates. The NIH criteria defines the disorder as having 1) hyperandrogenism and/or hyperandrogenemia, 2) oligoovulation and 3) the exclusion of other known disorders.

In GBD 2019, there was no new systematic review undertaken for PCOS, although prior to modelling the data used in GBD 2017 were reviewed and re-extracted. In addition to the diagnostic criteria, IHME also identified whether the cases of PCOS had been diagnosed by a physician or were self-reported. The data used in GBD 2019 were first extracted for GBD 2010, where a systematic review was conducted using the Ovid, MEDLINE, EMBASE, CINAHL, CAB abstracts, WHOLIS, ISGLE and PubMed databases. For example, the search strings used for PubMed were (“Polycystic Ovary Syndrome”[Mesh] OR “Polycystic Ovary Syndrome” OR “Sclerocystic Ovary Syndrome” OR “Sclerocystic Ovarian Degeneration” OR “Stein-Leventhal Syndrome” OR “Stein Leventhal Syndrome” OR “Sclerocystic Ovaries” OR “Sclerocystic Ovary”) AND (“Incidence”[Mesh] OR Incidence OR Incidences OR “Prevalence”[Mesh] OR Prevalence OR Prevalences).

Studies were excluded if they did not provide primary data on epidemiological parameters (e.g. reviews, commentaries and letters) or were clearly not representative of the population (e.g. only high-risk pregnant women). As well as the previously used data, GBD 2019 also incorporated claims and hospital administrative data from the United States of America (i.e. MarketScan), Philippines, Taiwan and Poland, resulting in a substantially larger data set than was available in previous GBD iterations.

### Data processing and disease model

Prior to modelling, age splitting was performed to ensure that all data matched the GBD defined age groups. In addition, although the NIH criteria was used as the reference definition, data using alternative definitions were adjusted using two MR-BRT (a meta-regression tool) models. Acceptable alternate definitions included the Rotterdam criteria, AE-PCOS criteria, self-reported PCOS, as well as clinical data.

The incidence and remission of PCOS were modelled using DisMod-MR 2.1. Incidence was set to only include women between menarche and menopause (i.e., those aged < 10 or > 55 were excluded). Remission up to 54 years old was constrained to a maximum value of 1 per 10 person-years, but after 54 there were no priors for remission. PCOS was not considered to be a cause of death, hence excess mortality was set to 0 and the non-fatal estimation process did not include a cause-specific mortality analysis. A decreasing slope, prior to the annual incidence starting at age 16, was used to facilitate matching the model with the highest annual incidence found among younger women (13–20 years old). The addition of clinical data enabled us to decrease the time span of data used to fit a particular year down to five years, instead of the previously used 20 year window. Additional details on the modelling of PCOS can be found in a previous publication^13^.

### Years lived with disability

The basis of the GBD disability weight survey assessments are lay descriptions of the sequelae, which highlight the major functional consequences and symptoms of PCOS^20^. Unfortunately, no PCOS specific health states were included in the GBD disability weights survey. The main sequelae of PCOS are infertility and hyperandrogenism/hirsutism, the latter of which was approximated with the health state of “disfigurement, level 1.” The NIH definition considers that all cases of PCOS have hyperandrogenism and hirsutism, and thus we assumed that 100% of PCOS cases would experience these sequelae.

DALY, which is a combination of the years of life lost due to premature mortality and the YLDs, is a standard metric used to quantify the burden of a disease^21^. However, in this case the YLD and DALY estimates were the same, as there was no evidence of PCOS-related mortality^13^. The YLDs for PCOS were estimated by multipling the appropriate disability weight by its point prevalence. All estimates were reported along with their corresponding 95% uncertainty intervals (UIs). Uncertainty was estimated from multiple sources, including input data, corrections of measurement error and estimates of residual non-sampling error, and by sampling 1,000 draws at each computational step. The UIs were defined as the 25th and 975th values of the ordered draws.

### Statistical analysis

The association between the burden of PCOS (i.e., YLDs) and SDI, for the 21 countries in the MENA region, were assessed using smoothing splines models^22^. The SDI ranges from 0 (less developed) to 1 (most developed) and is comprised of the: (1) lag-distributed income per capita, which is the gross domestic product per capita smoothed over the preceding decade; (2) average years of schooling for the population older than 15 years of age; and (3) total fertility rate under the age of 25. The statistical analyses were conducted using R software, version 3.5.2.

### Ethics approval and consent to participate

The present study was approved by the ethics committee of the Shahid Beheshti University of Medical Sciences (IR.SBMU.RETECH.REC.1400.861) and the Tabriz University of Medical Sciences (IR.TBZMED.REC.1400.596). All methods were performed in accordance with national guidelines and regulations.

## Results

### The Middle East and North Africa region

In 2019, there were 6,647,566 prevalent cases of PCOS in the MENA region, with an age-standardised point prevalence of 2079.7 per 100,000 women, which represents a 37.9% increase since 1990. PCOS accounted for 236,312 incident cases in 2019, with an age-standardised rate of 77.2 per 100,000 women, which has increased 33.7% since 1990 (Table 1). In 2019, the regional number of YLDs was 59,835, with an age-standardised rate of 18.7 YLDs per 100,000 women, an increase of 36.1% since 1990 (Table 1).

**Table 1:** Prevalent cases, incident cases and years lived with disability (YLDs) due to polycystic ovary syndrome in the Middle East and North Africa region in 2019 and the percentage change in the age- standardised rates (ASRs) per 100,000 women from 1990 to 2019 (Generated from data available from http://ghdx.healthdata.org/gbd-results-tool).

	Prevalence (95% UI)			Incidence (95% UI)			YLDs (95% UI)		
	Counts (2019)	ASRs (2019)	Pcs in ASRs 1990–2019	Counts (2019)	ASRs (2019)	Pcs in ASRs 1990–2019	Counts (2019)	ASRs (2019)	Pcs in ASRs 1990–2019
Middle East and North Africa	6,647,566 (4,445,554, 8,990,141)	2079.7 (1392, 2812.3)	37.9 (31.7, 45)	236,312 (158,280, 322,447)	77.2 (51.6, 105.4)	33.7 (27.7, 40.3)	59,835 (24,959, 121,070)	18.7 (7.8, 37.9)	36.1 (29.4, 43.4)
Afghanistan	216,686 (145,206, 298,254)	1213.1 (815.1, 1663.6)	50.1 (31, 72.6)	11,948 (8073, 16,278)	46.6 (31.6, 63.4)	49 (30, 71.6)	1951 (801, 3924)	10.8 (4.5, 21.6)	50.4 (31.3, 75.6)
Algeria	499,279 (326,272, 702,506)	2227.6 (1455.6, 3143.5)	51.4 (32.1, 69.6)	15,932 (10,376, 22,521)	85.6 (55.7, 120.8)	50.6 (31.2, 68.1)	4451 (1848, 9058)	19.9 (8.2, 40.4)	50.1 (30.6, 68.7)
Bahrain	16,239 (10,544, 22,020)	2477.5 (1610, 3370.1)	16 (2.8, 34.7)	448 (291, 609)	94.6 (61, 129.5)	15.9 (2.5, 34.6)	144 (59, 300)	22.2 (9.1, 45.6)	16.3 (1.7, 35.1)
Egypt	1,192,757 (801,347, 1,643,434)	2357.1 (1586.6, 3246.6)	29.4 (10.9, 46.9)	49,169 (32,675, 68,385)	90.4 (60.1, 125.3)	29.4 (9.9, 48.3)	10,824 (4445, 22,024)	21.3 (8.8, 43.4)	26.9 (8.5, 45.8)
Iran (Islamic Republic of)	1,011,263 (685,287, 1,360,965)	2166.2 (1465.3, 2924.5)	38.1 (31.4, 45.9)	28,008 (19,027, 37,687)	83 (56.2, 110.9)	38.5 (32.3, 46.4)	9319 (3927, 18,993)	20 (8.5, 40.7)	38.2 (31.3, 46.5)
Iraq	455,162 (301,674, 624,401)	2014.1 (1334.2, 2761.8)	13.4 (0, 30.4)	19,121 (12,617, 26,267)	77.1 (51, 106.7)	13.1 (-0.7, 30.3)	4100 (1742, 8360)	18 (7.7, 36.7)	13.7 (0.3, 30.6)
Jordan	122,519 (79,633, 172,846)	2111.3 (1373.8, 2976.7)	31.4 (15.1, 51)	5486 (3544, 7778)	81.1 (52.5, 114.8)	29.3 (12.9, 49.1)	1117 (480, 2281)	19.2 (8.2, 39.4)	31.7 (15, 51.8)
Kuwait	77,192 (51,529, 106,093)	2838.1 (1892.8, 3917.8)	21.5 (5.9, 41.2)	1566 (1037, 2186)	108.6 (71.7, 151.4)	21.7 (6.7, 41.8)	685 (288, 1396)	25.4 (10.7, 51.2)	21.3 (5.5, 40.5)
Lebanon	63,949 (43,564, 87,115)	2357.8 (1607, 3206.1)	32.6 (15.5, 52.1)	1860 (1238, 2573)	89.7 (60.4, 123.7)	32.5 (15.1, 51.4)	564 (235, 1142)	20.9 (8.8, 42)	32.6 (15.1, 52.1)
Libya	89,849 (58,840, 123,703)	2282.2 (1493.1, 3146.1)	12 (2.1, 26.6)	2805 (1827, 3863)	87.5 (56.6, 121.6)	11.2 (-0.1, 25.7)	804 (328, 1656)	20.5 (8.3, 42.3)	12.2 (1.1, 26.3)
Morocco	394,014 (258,695, 534,776)	2044.4 (1341.9, 2775.7)	37.6 (20.2, 58.8)	13,917 (9128, 18,931)	78.1 (51.2, 106.8)	36 (17.6, 55.9)	3545 (1481, 7243)	18.4 (7.7, 37.5)	36.6 (18.4, 58.8)
Oman	47,369 (30,703, 65,241)	2456.7 (1592.5, 3389.6)	74.1 (51.3, 102.5)	1436 (932, 1978)	94.1 (60.8, 130)	73.9 (51.6, 102.1)	426 (182, 879)	22 (9.4, 45.2)	73.9 (50.4, 105.2)
Palestine	48,156 (31,188, 67,563)	1903.2 (1234.2, 2659.6)	28.4 (12.8, 46)	2281 (1477, 3211)	72.9 (47.4, 102)	27.9 (10.1, 46.4)	435 (178, 893)	17.1 (7, 35.3)	28.2 (11.2, 46.9)
Qatar	25,366 (16,679, 35,252)	2748.1 (1811.4, 3821.7)	18.6 (5.7, 33.9)	634 (410, 903)	105.1 (68, 147.9)	18.6 (4.5, 35.2)	226 (94, 454)	24.6 (10.4, 49)	18.6 (5.6, 36)
Saudi Arabia	525,370 (345,147, 714,650)	2692 (1761.8, 3672.7)	45.7 (28.3, 68.8)	13,509 (8809, 18,607)	103 (66.9, 143)	45.6 (27.9, 68.2)	4710 (1973, 9612)	24.2 (10.1, 49.2)	45.4 (26.9, 67.6)
Sudan	384,497 (256,491, 533,120)	1796.5 (1207.7, 2483.6)	91.7 (65.8, 119.3)	17,976 (11,776, 24,965)	68.2 (44.8, 94.7)	89.2 (64.7, 116.7)	3370 (1413, 7043)	15.7 (6.5, 32.7)	90.3 (64.1, 120.9)
Syrian Arab Republic	163,305 (108,961, 230,894)	2043.9 (1363.2, 2874.6)	36.2 (19.2, 54.3)	7741 (5127, 10,654)	78.4 (51.8, 109.2)	35.4 (18.1, 53.8)	1451 (613, 2964)	18.1 (7.7, 37.1)	36.6 (18.6, 56.9)
Tunisia	129,760 (87,349, 180,477)	2109.2 (1422.4, 2930)	46.6 (28.2, 69.5)	3799 (2517, 5212)	80.9 (53.3, 111.4)	46.5 (28.3, 71.1)	1165 (501, 2400)	19 (8.3, 39.1)	43.8 (25.8, 65.5)
Turkey	898,978 (597,645, 1,229,210)	2026.3 (1350, 2773)	46.8 (29.7, 67.2)	26,634 (17,679, 36,724)	77.6 (51.3, 107.4)	45.3 (29.1, 67.2)	8006 (3375, 16,346)	18.1 (7.6, 37.1)	42.4 (23.7, 64.2)
United Arab Emirates	84,560 (56,514, 115,197)	2505.8 (1680.1, 3427.2)	34.8 (17.7, 51.8)	2009 (1343, 2812)	95.9 (64, 133.1)	34.7 (16.4, 51.6)	748 (314, 1512)	22.5 (9.4, 45.7)	34.8 (16.7, 52.8)
Yemen	194,542 (129,602, 267,496)	1231.2 (824, 1687.5)	27.5 (12.3, 42.7)	97,912 (6528, 13,443)	47.1 (31.7, 64.5)	26.7 (11.9, 42.3)	1735 (729, 3520)	10.9 (4.6, 22)	26.8 (10.7, 44.2)

### National level

**Figure 1:**
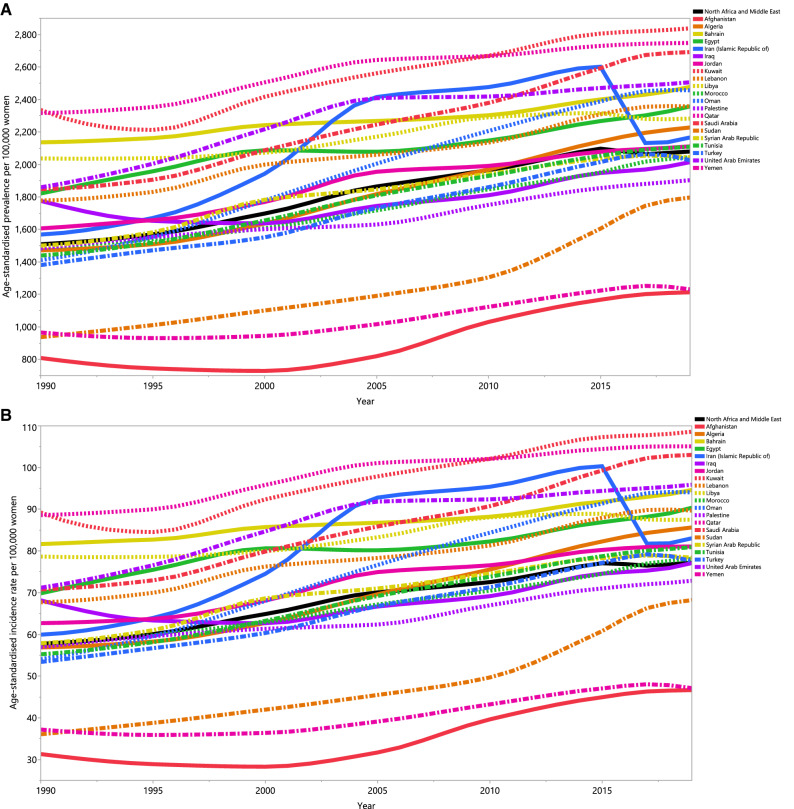

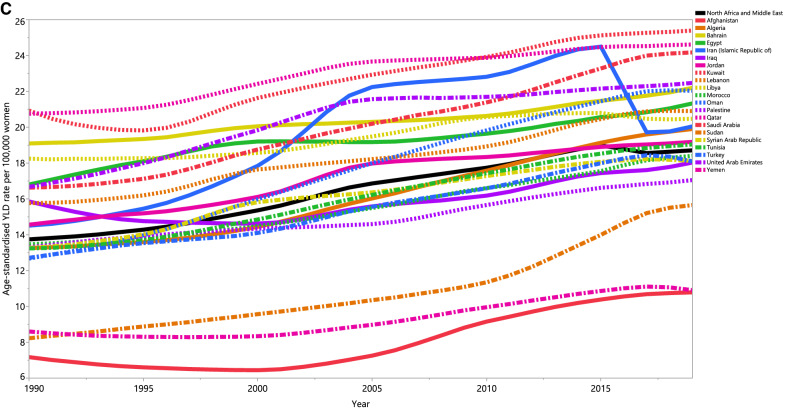
Age-standardised point prevalence (**A**), incidence (**B**), and YLDs (**C**) for polycystic ovary syndrome (per 100,000 population) in the Middle East and North Africa region from 1990 to 2019, by country. YLD = years lived with disability. (Generated from data available from http://ghdx.healthdata.org/gbd-results-tool).

In 2019, the age-standardised point prevalence of PCOS ranged from 1,213.1 to 2,838.1 (per 100,000 women) in the 21 countries and territories that comprise the MENA region. Kuwait [2,838.1 (95% UI: 1,892.8 to 3,917.8], Qatar [2,748.1 (95% UI: 1,811.4 to 3,821.7)] and Saudi Arabia [2,692.0 (95% UI: 1761.8 to 3672.7)] had the highest age-standardised point prevalence. In contrast, Afghanistan [1,213.1 (95% UI: (815.1 to 1,663.6)], Yemen [1,231.2 (95% UI: 824.0 to 1,687.5)] and Sudan [1,796.5 (95% UI: 1,207.7 to 2,483.6)] had the lowest (Fig. 1A and Table S1).

The age-standardised annual incidence rate of PCOS also varied substantially by country. In 2019, Kuwait [108.6 (95% UI: 71.7 to 151.4)], Qatar [105.1 (95% UI: 68.0 to 147.9)] and Saudi Arabia [103.0 (95% UI: 66.9 to 143.0)] had the highest age-standardised annual incidence rates. In contrast, Afghanistan [46.6 (95% UI: 31.6 to 63.4)], Yemen [47.1 (95% UI: 31.7 to 64.5)] and Sudan [68.2 (95% UI: (44.8 to 94.7)] had the lowest rates (Fig. 1B, Table S2).

In 2019, Kuwait [25.4 (95% UI: (10.7 to 51.2)], Qatar [24.6 (95% UI: 10.4 to 49.0)] and Saudi Arabia [24.2 (95% UI: 10.1 to 49.2)] had the highest age-standardised YLD rates for PCOS. In contrast, Afghanistan [10.8 (95% UI: 10.1 to 49.2)], Yemen [10.9 (95% UI: 4.6 to 22.0)] and Sudan [15.7 (95% UI: 6.5 to 32.7)] had the lowest rates (Fig. 1C and Table S3).

There were also substantial differences in the percentage change in the age-standardised point prevalence of PCOS over the measurement period (1990–2019). Sudan [91.7% (95% UI: 65.8 to 119.3)], Oman [71.4% (95% UI: 51.3 to 102.5)] and Algeria [51.4% (95% UI: 32.1 to 69.6)] showed the largest increases, while there were no countries which decreased during the measurement period (Fig. 1A and Table S1).

The percentage change in the age-standardised annual incidence rate of PCOS (from 1990–2019) also differed substantially between countries. The largest increases were observed in Sudan [89.2% (95% UI: 64.7 to 116.7)], Oman [73.9% (95% UI: 51.6 to 102.1)] and Algeria [50.6% (95% UI: 31.2 to 68.1)]. There were no decreases observed in any of the MENA countries during the measurement period (Fig. 1B and Table S2).

The percentage change in the age-standardised YLD rate of PCOS, from 1990 to 2019, also differed substantially by country. The largest increases were seen in Sudan [90.3% (95% UI: 64.1 to 120.9)], Oman [73.9% (95% UI: 50.4 to 105.2)] and Afghanistan [50.4% (95% UI: 31.3 to 75.6)]. There were no decreases observed in any of the MENA countries over the measurement period (Fig. 1C and Table S3).

### Age pattern

**Figure 2:**
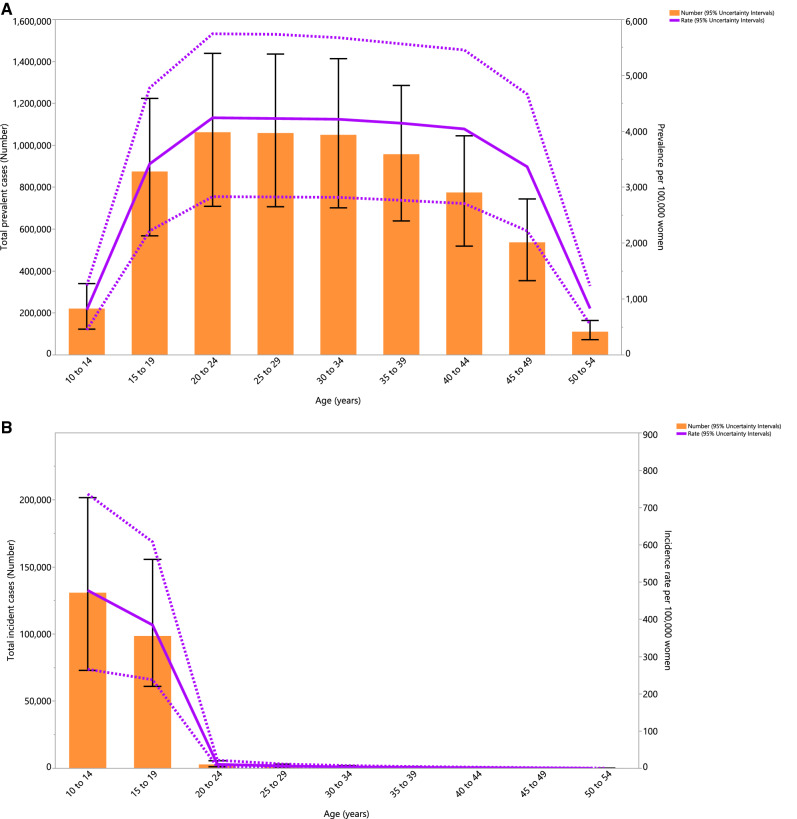

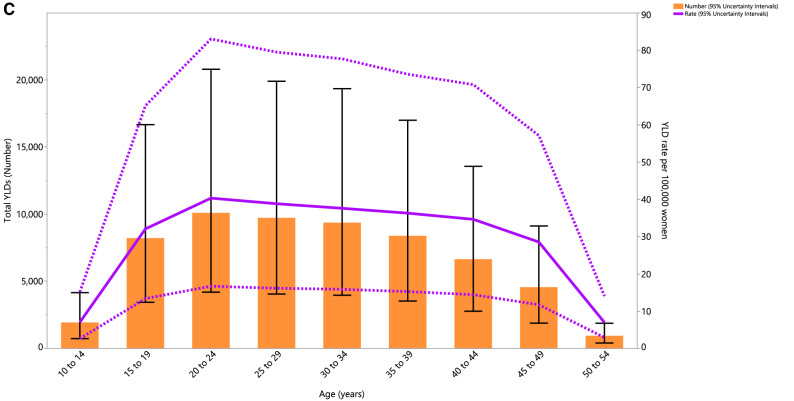
Number of prevalent cases and prevalence (**A**), number of incident cases and incidence rate (**B**), and the number of YLDs and YLD rate (**C**) for polycystic ovary syndrome (per 100,000 population) in the Middle East and North Africa region, by age, in 2019; Dotted lines indicate 95% upper and lower uncertainty intervals, respectively. The black solid vertical lines represent the 95% uncertainty interval for the number of prevalent cases, incident cases, and YLDs. YLD = years lived with disability. (Generated from data available from http://ghdx.healthdata.org/gbd-results-tool).

In 2019, the number of prevalent cases and the point prevalence of PCOS (per 100,000 population) increased with advancing age, and both of which peaked in the 20–24 age group. The number of cases and the point prevalence were both highest in women of reproductive age, but both decreased rapidly from 45 years of age (Fig. 2A). Also, the annual incidence rate and number of incident cases peaked in the 10–14 age group (Fig. 2B). Similar to the prevalence of PCOS, the YLD rate and number of YLDs peaked in the 20–24 age group (Fig. 2C).

### Burden by socio-demographic index (SDI)

**Figure 3:**
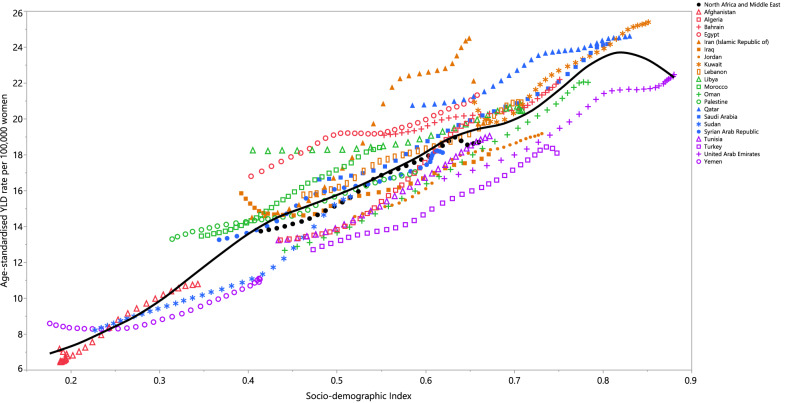
Age-standardised YLD rates of polycystic ovary syndrome for the 21 countries and territories of the Middle East and North African region by Socio-demographic Index, 1990–2019; Expected values based on the Socio-demographic Index and the disease rates in all locations are shown as the black line. Countries above the solid line (e.g. Qatar and Morocco) had higher than expected burdens, while those below the line (e.g. Turkey and Jordan) had lower than expected burdens. Thirty points are plotted for each GBD region and show the observed age-standardised YLD rates from 1990 to 2019 for that region. YLD = years lived with disability. (Generated from data available from http://ghdx.healthdata.org/gbd-results-tool).

In 2019, the age-standardised YLD rate increased with increasing SDI. In other words, there was a positive association between SDI and the age-standardised YLD rate due to PCOS. Countries such as Libya, Egypt, Qatar, Iran, Saudi Arabia, Kuwait, Bahrain and Morroco had higher than expected age-standardised YLDs, while countries like Turkey, Oman, Tunisia and Jordan had lower than expected age-standardised YLDs (Fig. 3).

## Discussion

The present study showed that there were great differences in the age-standardised prevalence of PCOS in the MENA countries, with the lowest estimate being found in Afghanistan and the highest in Kuwait. Furthermore, the largest burdens of PCOS were found in the third and fourth decades of life, and the burden increased with increasing sociodemographic development.

The overall increase in PCOS, that was found in this study, might be due to the inherited nature of this syndrome, along with changes in the diagnostic criteria over the last few decades. For example, the estimated incidence of PCOS is significantly higher when using the Rotterdam criteria, and this remains the most widely used diagnostic criteria across the world^23^. Moreover, it has been claimed that approximately 70% of those with PCOS remain undiagnosed, even after visiting several medical practitioners^24,25^, indicating that the incidence of this condition is likely to be greatly underestimated.

PCOS is also a familial condition, as clinical studies have identified an autosomal dominant gene as being important^26^. However, the inter-country differences suggest that both genetic and enviromental factors have an important role in the development of PCOS. Previous studies have reported decreases in the incidence and DALYs associated with PCOS in the MENA region, but this was likely due to lower detection rates, as a consequence of the lower resource availability in the low-to middle-income countries in this region^27^. Furthermore, the same studies have shown that this decrease was exacerbated by international conflicts and the emigration of many experienced healthcare workers^27^. Therefore, the findings of the current study may reflect higher detection rates, more advanced healthcare infrastructures and better primary healthcare in these countries. Furthermore, the lowest age-standardised incidence rates and DALYs for PCOS were observed in the low-SDI countries, which again is likely to be indicative of the lower detection rates in these countries. For example, ultrasonography, which is commonly used to diagnose PCOS, is limited in Africa, both in terms of its availability and its affordability^28^. A lack of awareness about PCOS by health professionals may be another contributing factor^29^. The low incidence rates in countries like Afghanistan and Yemen can also be explained using the aforementioned reasons.

The YLD rate of PCOS in the MENA region was higher than the global average (18.7 vs. 14.7 per 100,000 population). Furthermore, the age-standardised YLD rate increased at both the global and regional levels, with a higher increase being found in the MENA region (36.1% vs. 29.9%). The large increase in the incidence of PCOS may be related to: population growth and aging, resource availability, healthcare access, health awareness, and obesity^9^. In particular, the recent increases in the obesity rates are likely to have contributed to the increased incidence of PCOS, as previous research has found the prevalence of PCOS to be 2–3 times higher amongst women classified as obese, than among women with a healthy body mass index (BMI)^30^.

Worldwide, obesity has more than doubled since 1980. Research on the relationship between economic development and health has identified several competing structural explanations for the rising BMI, including globalization, economic development and the changing role of women in society. Globalization, particularly trade liberalization, has contributed to the global weight gain by facilitating the diffusion of obesogenic products into low and middle income countries, such as sugar-sweetened beverages and packaged foods^31^.

As recent studies have shown, obesity is significantly higher among women than among men in Morocco and Tunisia (22.7% vs. 6.7% in Tunisia and 18% vs. 5.7% in Morocco), and the prevalence among women has tripled over the last 20 years^32^. The proportion of individuals who are overweight increases with age and seems to take hold in adolescence, particularly among girls. In Tunisia, 9.1% of adolescent girls are at risk of becoming overweight (BMI/age > or = 85th percentile)^32^. Moreover, the prevalence of overweight and obesity exacerbates the reproductive and metabolic manifestations of PCOS. The symptoms of PCOS often begin during adolescence, and the rising prevalence of peripubertal obesity has prompted concern that the prevalence and severity of adolescent PCOS is increasing in parallel. Recent studies have suggested that such girls are indeed at risk of developing PCOS. Obesity may impact the risk of PCOS via insulin resistance and compensatory hyperinsulinemia, which augments ovarian/adrenal androgen production and suppresses sex hormone–binding globulin (SHBG), thereby increasing androgen bioavailability^33^.

The highest number of PCOS incident cases were found among women aged 10–14 years old and the highest number of YLDs were found in the 20–24 age group. Consistent with our findings in the MENA region, the global incident cases and DALYs were highest in the 15–19 and 25–29 age groups, respectively. The slight discrepancy might be due to the difficulty in diagnosing PCOS during adolescence, since the symptoms of PCOS overlap with some of the physiological changes observed during adolescence, the low availability of appropriate diagnostic techniques in the MENA region (e.g., transvaginal ultrasound), and the use of different criteria for diagnosis^34,35^.

Our results revealed a generally positive association between the burden of PCOS and SDI, suggesting that the rates of PCOS increase with higher income per capita, higher educational attainment and a lower total fertility rate. However, over the period 2007–2017, the global age-standardised DALY rate of PCOS had a non-linear association with SDI, peaking at an SDI of 0.66^9^. The higher burden of PCOS in high SDI countries may be due to more advanced healthcare systems, which result in higher detection rates. An increased life expectancy, resulting in population growth and aging, may also contribute to the higher PCOS burden in developed countries. Further studies utilising decomposition analyses are necessary to determine the proportion of the attributable burden which is related to population growth and aging. Moreover, the better access to nutrition for those living with PCOS in high SDI countries could also increase the survival of women with hyperinsulinemia/insulin resistance during seasonal or permanent periods of undernutrition, meaning that the prevalence of PCOS will increase in these countries^36^.

In order to reduce the incidence and burden of PCOS comorbidities, preventive programs should focus on weight assessment and waist circumference measurements every 6–12 months, the evaluation of glycated hemoglobin (i.e. HbA1C) every three years and the use of oral glucose tolerance tests (OGTT) every 1–3 years amongst women susceptible to type 2 diabetes mellitus^37^. Moreover, regular assessments and treatment of hypertension, dyslipidemia, psychological stress and mental disorders should be undertaken in patients diagnosed with PCOS^37^.

### Strength and limitations of this study

The present study is the first to use a modeling strategy to estimate the burden of PCOS in the MENA region and to report the burden for all 21 countries, according to a number of demographic and non-demographic variables. However, we also acknowledge that our study has several limitations. Firstly, a major issue for all GBD studies is shortcomings in the data registries and data collection systems, which may lead to an under- or over-estimation of the PCOS burden. Also, differences in the PCOS criteria used across the MENA countries might hinder an accurate comparison between the individual countries. Secondly, the effects of the COVID-19 pandemic on the PCOS burden were not evaluated in the present study. Evidence shows that there is bidirectional association between COVID-19 and PCOS, which can affect the quality of life and the prevalence of patients with PCOS^38,39^.

## Conclusions

The burden of PCOS is increasing at both the regional and country level in the MENA region, and this burden is higher than the global average. The incidence of PCOS increased in all countries located in the MENA region, which highlights the need for the implementation of cost-effective preventive programs focusing on addressing risk factors, especially for women in their third decade of life, and in MENA countries with higher SDIs. Further studies are recommended to provide data on the effects that the COVID-19 pandemic has had on the burden of PCOS. Moreover, the PCOS-related health facilities and health policies of the individual countries should be evaluated in future research.

## Supplementary Information

Supplementary Information 1.

Supplementary Information 2.

Supplementary Information 3.

## Data Availability

The data used for these analyses are all publicly available at http://ghdx.healthdata.org/gbd-results-tool. This study is based on publicly available data and solely reflects the opinion of its authors and not that of the Institute for Health Metrics and Evaluation.
