# Supplementary material for: Burden of polycystic ovary syndrome in the Middle East and North Africa region, 1990–2019
Source: Sci Rep. 2022 Apr 29;12:7039. doi: 10.1038/s41598-022-11006-0 (PMC9052181; doi:10.1038/s41598-022-11006-0)
Supplement: Supplementary file 1 — Supplementary Information 1. [file 41598_2022_11006_MOESM1_ESM.docx]

| **Table S1: Prevalence of polycystic ovary syndrome in 1990 and 2019 and the percentage change in the age-standardised rates (ASRs) per 100,000 women in the Middle East and North Africa region**  **(Generated from data available from http://ghdx.healthdata.org/gbd-results-tool)** | | | | | |
| --- | --- | --- | --- | --- | --- |
|  | **1990** | | **2019** | | **Percentage change in ASRs per 100,000** |
|  | **No (95% UI)** | **ASRs per 100,000 (95% UI)** | **No (95% UI)** | **ASRs per 100,000 (95% UI)** |  |
| **Middle East and North Africa** | **2426961 (1638015 , 3262014)** | **1508.4 (1018.2 , 2026.4)** | **6647566 (4445554 , 8990141)** | **2079.7 (1392 , 2812.3)** | **37.9 (31.7 , 45)** |
| **Afghanistan** | **41089 (26841 , 56864)** | **808.4 (528.9 , 1110.1)** | **216686 (145206 , 298254)** | **1213.1 (815.1 , 1663.6)** | **50.1 (31 , 72.6)** |
| **Algeria** | **174972 (114151 , 240669)** | **1471.3 (965.1 , 2007.5)** | **499279 (326272 , 702506)** | **2227.6 (1455.6 , 3143.5)** | **51.4 (32.1 , 69.6)** |
| **Bahrain** | **5015 (3348 , 6901)** | **2136.2 (1429.1 , 2908.7)** | **16239 (10544 , 22020)** | **2477.5 (1610 , 3370.1)** | **16 (2.8 , 34.7)** |
| **Egypt** | **484498 (318706 , 667424)** | **1821.2 (1200 , 2506.4)** | **1192757 (801347 , 1643434)** | **2357.1 (1586.6 , 3246.6)** | **29.4 (10.9 , 46.9)** |
| **Iran (Islamic Republic of)** | **415826 (279207 , 563608)** | **1568.5 (1059.7 , 2118)** | **1011263 (685287 , 1360965)** | **2166.2 (1465.3 , 2924.5)** | **38.1 (31.4 , 45.9)** |
| **Iraq** | **138294 (91683 , 188988)** | **1775.8 (1178 , 2415.5)** | **455162 (301674 , 624401)** | **2014.1 (1334.2 , 2761.8)** | **13.4 (0 , 30.4)** |
| **Jordan** | **27455 (18222 , 38193)** | **1606.3 (1072.7 , 2213.3)** | **122519 (79633 , 172846)** | **2111.3 (1373.8 , 2976.7)** | **31.4 (15.1 , 51)** |
| **Kuwait** | **20058 (13425 , 27695)** | **2335.3 (1563.9 , 3228.5)** | **77192 (51529 , 106093)** | **2838.1 (1892.8 , 3917.8)** | **21.5 (5.9 , 41.2)** |
| **Lebanon** | **27519 (18457 , 37878)** | **1778.2 (1192.2 , 2447.4)** | **63949 (43564 , 87115)** | **2357.8 (1607 , 3206.1)** | **32.6 (15.5 , 52.1)** |
| **Libya** | **36793 (24091 , 50347)** | **2036.9 (1339.4 , 2776.6)** | **89849 (58840 , 123703)** | **2282.2 (1493.1 , 3146.1)** | **12 (2.1 , 26.6)** |
| **Morocco** | **189956 (128075 , 258455)** | **1485.7 (1002.8 , 2023.3)** | **394014 (258695 , 534776)** | **2044.4 (1341.9 , 2775.7)** | **37.6 (20.2 , 58.8)** |
| **Oman** | **9817 (6530 , 13464)** | **1410.8 (939.6 , 1928.6)** | **47369 (30703 , 65241)** | **2456.7 (1592.5 , 3389.6)** | **74.1 (51.3 , 102.5)** |
| **Palestine** | **13264 (8789 , 18530)** | **1482.8 (985.9 , 2059.7)** | **48156 (31188 , 67563)** | **1903.2 (1234.2 , 2659.6)** | **28.4 (12.8 , 46)** |
| **Qatar** | **3713 (2440 , 5244)** | **2316.9 (1527.2 , 3255.2)** | **25366 (16679 , 35252)** | **2748.1 (1811.4 , 3821.7)** | **18.6 (5.7 , 33.9)** |
| **Saudi Arabia** | **123981 (80367 , 168902)** | **1848 (1201.2 , 2507.9)** | **525370 (345147 , 714650)** | **2692 (1761.8 , 3672.7)** | **45.7 (28.3 , 68.8)** |
| **Sudan** | **88712 (58437 , 121540)** | **937 (619.8 , 1284)** | **384497 (256491 , 533120)** | **1796.5 (1207.7 , 2483.6)** | **91.7 (65.8 , 119.3)** |
| **Syrian Arab Republic** | **84784 (55951 , 118203)** | **1500.2 (992.6 , 2066.4)** | **163305 (108961 , 230894)** | **2043.9 (1363.2 , 2874.6)** | **36.2 (19.2 , 54.3)** |
| **Tunisia** | **59898 (39379 , 82449)** | **1439.1 (946.4 , 1997.8)** | **129760 (87349 , 180477)** | **2109.2 (1422.4 , 2930)** | **46.6 (28.2 , 69.5)** |
| **Turkey** | **412515 (274956 , 566944)** | **1380.6 (924.7 , 1897)** | **898978 (597645 , 1229210)** | **2026.3 (1350 , 2773)** | **46.8 (29.7 , 67.2)** |
| **United Arab Emirates** | **12971 (8375 , 18037)** | **1859.1 (1198.9 , 2574.3)** | **84560 (56514 , 115197)** | **2505.8 (1680.1 , 3427.2)** | **34.8 (17.7 , 51.8)** |
| **Yemen** | **54199 (35854 , 73168)** | **965.3 (642.6 , 1292.9)** | **194542 (129602 , 267496)** | **1231.2 (824 , 1687.5)** | **27.5 (12.3 , 42.7)** |
