# Supplementary material for: Burden of polycystic ovary syndrome in the Middle East and North Africa region, 1990–2019
Source: Sci Rep. 2022 Apr 29;12:7039. doi: 10.1038/s41598-022-11006-0 (PMC9052181; doi:10.1038/s41598-022-11006-0)
Supplement: Supplementary file 2 — Supplementary Information 2. [file 41598_2022_11006_MOESM2_ESM.docx]

| **Table S2: Incidence of polycystic ovary syndrome in 1990 and 2019 and the percentage change in the age-standardised rates (ASRs) per 100,000 women in the Middle East and North Africa region**  **(Generated from data available from http://ghdx.healthdata.org/gbd-results-tool)** | | | | | |
| --- | --- | --- | --- | --- | --- |
|  | **1990** | | **2019** | | **Percentage change in ASRs per 100,000** |
|  | **No (95% UI)** | **ASRs per 100,000 (95% UI)** | **No (95% UI)** | **ASRs per 100,000 (95% UI)** |  |
| **Middle East and North Africa** | **127161 (85004 , 172459)** | **57.7 (38.9 , 77.8)** | **236312 (158280 , 322447)** | **77.2 (51.6 , 105.4)** | **33.7 (27.7 , 40.3)** |
| **Afghanistan** | **2435 (1619 , 3333)** | **31.3 (20.9 , 42.4)** | **11948 (8073 , 16278)** | **46.6 (31.6 , 63.4)** | **49 (30 , 71.6)** |
| **Algeria** | **9646 (6234 , 13322)** | **56.9 (37.1 , 78.2)** | **15932 (10376 , 22521)** | **85.6 (55.7 , 120.8)** | **50.6 (31.2 , 68.1)** |
| **Bahrain** | **195 (127 , 271)** | **81.7 (53.5 , 112.9)** | **448 (291 , 609)** | **94.6 (61 , 129.5)** | **15.9 (2.5 , 34.6)** |
| **Egypt** | **23588 (15339 , 32830)** | **69.8 (45.4 , 96.8)** | **49169 (32675 , 68385)** | **90.4 (60.1 , 125.3)** | **29.4 (9.9 , 48.3)** |
| **Iran (Islamic Republic of)** | **22821 (15267 , 30931)** | **59.9 (40.7 , 80.8)** | **28008 (19027 , 37687)** | **83 (56.2 , 110.9)** | **38.5 (32.3 , 46.4)** |
| **Iraq** | **8034 (5314 , 10909)** | **68.2 (45.3 , 92.5)** | **19121 (12617 , 26267)** | **77.1 (51 , 106.7)** | **13.1 (-0.7 , 30.3)** |
| **Jordan** | **1648 (1081 , 2311)** | **62.7 (41.4 , 87.9)** | **5486 (3544 , 7778)** | **81.1 (52.5 , 114.8)** | **29.3 (12.9 , 49.1)** |
| **Kuwait** | **775 (512 , 1090)** | **89.2 (59.2 , 124.5)** | **1566 (1037 , 2186)** | **108.6 (71.7 , 151.4)** | **21.7 (6.7 , 41.8)** |
| **Lebanon** | **1220 (808 , 1699)** | **67.7 (44.8 , 94.4)** | **1860 (1238 , 2573)** | **89.7 (60.4 , 123.7)** | **32.5 (15.1 , 51.4)** |
| **Libya** | **2338 (1516 , 3195)** | **78.7 (51.2 , 107.5)** | **2805 (1827 , 3863)** | **87.5 (56.6 , 121.6)** | **11.2 (-0.1 , 25.7)** |
| **Morocco** | **9392 (6270 , 12828)** | **57.4 (38.5 , 78.1)** | **13917 (9128 , 18931)** | **78.1 (51.2 , 106.8)** | **36 (17.6 , 55.9)** |
| **Oman** | **574 (370 , 803)** | **54.1 (35.6 , 74.5)** | **1436 (932 , 1978)** | **94.1 (60.8 , 130)** | **73.9 (51.6 , 102.1)** |
| **Palestine** | **771 (508 , 1079)** | **57 (37.9 , 79.5)** | **2281 (1477 , 3211)** | **72.9 (47.4 , 102)** | **27.9 (10.1 , 46.4)** |
| **Qatar** | **148 (97 , 210)** | **88.6 (58.3 , 124.4)** | **634 (410 , 903)** | **105.1 (68 , 147.9)** | **18.6 (4.5 , 35.2)** |
| **Saudi Arabia** | **6896 (4457 , 9504)** | **70.8 (46.1 , 97.3)** | **13509 (8809 , 18607)** | **103 (66.9 , 143)** | **45.6 (27.9 , 68.2)** |
| **Sudan** | **4696 (3128 , 6444)** | **36.1 (24.3 , 49.1)** | **17976 (11776 , 24965)** | **68.2 (44.8 , 94.7)** | **89.2 (64.7 , 116.7)** |
| **Syrian Arab Republic** | **5184 (3373 , 7235)** | **57.9 (38.3 , 79.9)** | **7741 (5127 , 10654)** | **78.4 (51.8 , 109.2)** | **35.4 (18.1 , 53.8)** |
| **Tunisia** | **2895 (1887 , 4008)** | **55.2 (36.2 , 76.2)** | **3799 (2517 , 5212)** | **80.9 (53.3 , 111.4)** | **46.5 (28.3 , 71.1)** |
| **Turkey** | **20071 (13336 , 27891)** | **53.4 (35.6 , 74)** | **26634 (17679 , 36724)** | **77.6 (51.3 , 107.4)** | **45.3 (29.1 , 67.2)** |
| **United Arab Emirates** | **522 (337 , 730)** | **71.2 (45.9 , 98.4)** | **2009 (1343 , 2812)** | **95.9 (64 , 133.1)** | **34.7 (16.4 , 51.6)** |
| **Yemen** | **3226 (2121 , 4373)** | **37.2 (24.8 , 49.5)** | **9792 (6528 , 13443)** | **47.1 (31.7 , 64.5)** | **26.7 (11.9 , 42.3)** |
