# Supplementary material for: Burden of polycystic ovary syndrome in the Middle East and North Africa region, 1990–2019
Source: Sci Rep. 2022 Apr 29;12:7039. doi: 10.1038/s41598-022-11006-0 (PMC9052181; doi:10.1038/s41598-022-11006-0)
Supplement: Supplementary file 3 — Supplementary Information 3. [file 41598_2022_11006_MOESM3_ESM.docx]

| **Table S3: Years lived with disability (YLDs) due to polycystic ovary syndrome in 1990 and 2019, and the percentage change in the age-standardised rates (ASRs) per 100,000 women in the Middle East and North Africa region**  **(Generated from data available from http://ghdx.healthdata.org/gbd-results-tool)** | | | | | |
| --- | --- | --- | --- | --- | --- |
|  | **1990** | | **2019** | | **Percentage change in ASRs per 100,000** |
|  | **No (95% UI)** | **ASRs per 100,000 (95% UI)** | **No (95% UI)** | **ASRs per 100,000 (95% UI)** |  |
| **Middle East and North Africa** | **22309 (9543 , 45143)** | **13.7 (5.9 , 27.8)** | **59835 (24959 , 121070)** | **18.7 (7.8 , 37.9)** | **36.1 (29.4 , 43.4)** |
| **Afghanistan** | **368 (155 , 746)** | **7.2 (3 , 14.5)** | **1951 (801 , 3924)** | **10.8 (4.5 , 21.6)** | **50.4 (31.3 , 75.6)** |
| **Algeria** | **1592 (650 , 3298)** | **13.3 (5.4 , 27.6)** | **4451 (1848 , 9058)** | **19.9 (8.2 , 40.4)** | **50.1 (30.6 , 68.7)** |
| **Bahrain** | **45 (19 , 92)** | **19.1 (7.9 , 39.2)** | **144 (59 , 300)** | **22.2 (9.1 , 45.6)** | **16.3 (1.7 , 35.1)** |
| **Egypt** | **4501 (1928 , 9189)** | **16.8 (7.2 , 34.4)** | **10824 (4445 , 22024)** | **21.3 (8.8 , 43.4)** | **26.9 (8.5 , 45.8)** |
| **Iran (Islamic Republic of)** | **3889 (1634 , 7901)** | **14.5 (6.1 , 29.2)** | **9319 (3927 , 18993)** | **20 (8.5 , 40.7)** | **38.2 (31.3 , 46.5)** |
| **Iraq** | **1247 (508 , 2523)** | **15.9 (6.6 , 32)** | **4100 (1742 , 8360)** | **18 (7.7 , 36.7)** | **13.7 (0.3 , 30.6)** |
| **Jordan** | **253 (106 , 511)** | **14.6 (6.1 , 29.4)** | **1117 (480 , 2281)** | **19.2 (8.2 , 39.4)** | **31.7 (15 , 51.8)** |
| **Kuwait** | **181 (74 , 364)** | **20.9 (8.6 , 41.8)** | **685 (288 , 1396)** | **25.4 (10.7 , 51.2)** | **21.3 (5.5 , 40.5)** |
| **Lebanon** | **245 (103 , 501)** | **15.8 (6.6 , 32.2)** | **564 (235 , 1142)** | **20.9 (8.8 , 42)** | **32.6 (15.1 , 52.1)** |
| **Libya** | **333 (139 , 668)** | **18.2 (7.6 , 36.8)** | **804 (328 , 1656)** | **20.5 (8.3 , 42.3)** | **12.2 (1.1 , 26.3)** |
| **Morocco** | **1736 (738 , 3536)** | **13.5 (5.8 , 27.4)** | **3545 (1481 , 7243)** | **18.4 (7.7 , 37.5)** | **36.6 (18.4 , 58.8)** |
| **Oman** | **89 (37 , 180)** | **12.7 (5.3 , 25.5)** | **426 (182 , 879)** | **22 (9.4 , 45.2)** | **73.9 (50.4 , 105.2)** |
| **Palestine** | **120 (49 , 245)** | **13.3 (5.5 , 26.9)** | **435 (178 , 893)** | **17.1 (7 , 35.3)** | **28.2 (11.2 , 46.9)** |
| **Qatar** | **33 (14 , 69)** | **20.8 (8.5 , 42.5)** | **226 (94 , 454)** | **24.6 (10.4 , 49)** | **18.6 (5.6 , 36)** |
| **Saudi Arabia** | **1127 (460 , 2326)** | **16.6 (6.8 , 34.1)** | **4710 (1973 , 9612)** | **24.2 (10.1 , 49.2)** | **45.4 (26.9 , 67.6)** |
| **Sudan** | **787 (330 , 1647)** | **8.2 (3.4 , 17)** | **3370 (1413 , 7043)** | **15.7 (6.5 , 32.7)** | **90.3 (64.1 , 120.9)** |
| **Syrian Arab Republic** | **759 (313 , 1532)** | **13.3 (5.4 , 27.1)** | **1451 (613 , 2964)** | **18.1 (7.7 , 37.1)** | **36.6 (18.6 , 56.9)** |
| **Tunisia** | **556 (238 , 1138)** | **13.2 (5.7 , 26.8)** | **1165 (501 , 2400)** | **19 (8.3 , 39.1)** | **43.8 (25.8 , 65.5)** |
| **Turkey** | **3828 (1635 , 7788)** | **12.7 (5.4 , 25.9)** | **8006 (3375 , 16346)** | **18.1 (7.6 , 37.1)** | **42.4 (23.7 , 64.2)** |
| **United Arab Emirates** | **117 (47 , 235)** | **16.7 (6.7 , 33.2)** | **748 (314 , 1512)** | **22.5 (9.4 , 45.7)** | **34.8 (16.7 , 52.8)** |
| **Yemen** | **487 (205 , 992)** | **8.6 (3.6 , 17.2)** | **1735 (729 , 3520)** | **10.9 (4.6 , 22)** | **26.8 (10.7 , 44.2)** |
